# Outcome of surgical management for rhegmatogenous retinal detachment in Behçet’s disease

**DOI:** 10.1186/1471-2415-14-61

**Published:** 2014-05-02

**Authors:** Sherif A Dabour, Manar A Ghali

**Affiliations:** 1 Department of Ophthalmology, Zagazig University, Zagazig, Egypt

**Keywords:** Rhegmatogenous retinal detachment, Behçet’s disease, Intravitreal injection, Triamcinolone acetonide, Scleral buckling

## Abstract

**Background:**

The purpose of the current study is to evaluate the surgical outcome for rhegmatogenous retinal detachment (RD) associated with Behçet’s disease (BD).

**Methods:**

The current retrospective study included all patients operated for rhegmatogenous RD associated with BD in our institution from June 2007 to June 2012. Surgical repair was done either by scleral buckling (SB) or pars plana vitrectomy (PPV) according to the topography and clinical criteria of the detachment.

**Results:**

The current study included 7 eyes of 7 patients (6 males and one female). The mean age was 34.3 ± 4.9 years and all patients showed systemic features of BD. In 3 eyes, intravitreal triamcinolone acetonide (IVTA) was injected within 8 weeks prior to the occurrence of rhegmatogenous RD. Five eyes were treated with SB (segmental buckle in 4 cases and encircling buckle in one case) and 2 cases were treated by PPV. One case was initially treated by SB but showed recurrence of RD which was surgically repaired by PPV with successful closure of the retinal break. The retina was successfully reattached in all cases at the end of follow up period (22.0 ± 6.7 months).

**Conclusions:**

Rhegmatogenous RD in BD can be effectively treated by scleral buckling in selected cases and PPV in more complex cases. Intravitreal injections may be a precipitating factor for rhegmatogenous RD.

## Background

Behçet's disease (BD) is multisystem disease characterized by recurrent attacks of vasculitis involving small, medium and large blood vessels of unknown cause [1]. The hallmark of BD is the recurrent episode of painful ulcers in the mouth and genitalia. BD often affects other systems in the body leading to skin lesions, arthritis and less frequently neurological, gastrointestinal and cardiovascular manifestations. BD is seen more commonly around the Mediterranean region, including Egypt, and the Far East [1,2].

Ocular involvement of BD is usually bilateral and typically presents during the working age group between the second and fourth decade. Ocular complications may occur in up to 95% of males and 70% of females with dramatic impact on vision. They include relapsing remitting panuveitis, retinal obliterative and necrotizing vasculitis, vitritis, vitreous hemorrhage, serous macular detachment, and rarely rhegmatogenous retinal detachment [2-6].

Pars plana vitrectomy has been reported to treat several posterior segment complications in patients with BD including vitreous hemorrhage, vitreous opacities, macular hole, and epiretinal membrane [7-12]. However there is only limited data on the surgical management of rhegmatogenous retinal detachment in eyes with BD [13,14].

In the study herein, we described a series of seven eyes with BD presented with rhegmatogenous RD with focus on the clinical course and visual outcome with surgical intervention.

## Methods

We retrospectively reviewed the medical records of patients who underwent surgical intervention for rhegmatogenous retinal detachment associated with BD during the period from June 2007 to June 2012. The Zagazig University Institutional Review Board (ZUIRB) approved this study, which adhered to the tenets of the Declaration of Helsinki.

Behçet’s disease was diagnosed according to the criteria of the international study group for Behçet’s disease (ISG) [15]. BD was considered if the subject has recurrent oral ulceration in addition to two of the following features; eye lesions, genital ulcers, skin lesions and positive pathergy test. All patients were on oral immunomodulatory therapy (Methotrexate or Cyclosporine or Azathioprine) with or without oral corticosteroids.

All subjects underwent a comprehensive ocular examination, including best-corrected visual acuity (VA) measurement (and corresponding log MAR conversion values), slit lamp examination, intraocular pressure measurement, and dilated color fundus photography (TRC50DX; Topcon Corp., Tokyo, Japan). Ultrasonographic evaluation (Sonomed, EZ scan AB5500+, New York, USA) was performed in all eyes just before surgical intervention.

Topical steroid eye drops (prednisolone acetate 1%) and systemic steroid (prednisolone 1 mg/Kg/day) were used for all patients prior to surgery. Eyes with single breaks with relatively healthy vitreous in the absence of any traction were treated by segmental scleral buckling procedure. Scleral buckling was combined with cryotherapy application and drainage of subretinal fluid. PPV was performed in eyes with more than one break or eyes with dense vitreous opacities or eyes that developed PVR.

All surgical interventions were done under general anesthesia. Conventional 3 port pars plana vitrectomy (PPV) procedure using 20-gauge vitrectomy system coupled with non-contact wide field viewing system (BIOM, OCULUS Optikgeräte GmbH, Wetzlar, Germany) with additional chandelier light when needed. During the surgery, the aim was to remove all vitreous as much as possible, all epiretinal membranes, and complete shaving of the vitreous base. In eyes operated with PPV, silicon oil tamponade was used at the end of the procedure. In all eyes, triamcinolone acetonide 40 mg (Kenacort-A, Bristol-Myers Squibb, 40 mg/1 ml) was injected into the subtenon space at the end of the surgery. All patients were hospitalized for 3–5 days postoperatively according to the course of the disease. The systemic corticosteroid therapy was tapered postoperatively according to the severity of the inflammation.

## Results

In the current study, seven eyes of 7 patients (6 (85.7%) males and (14.3%) female) with rhegamtougenous RD associated with BD were included. The mean age was 34.3 ± 4.9 years (range from 28 to 42 years). The mean time elapsed since diagnosis of BD was 5.4 ± 1.6 years (range from 3 to 7 years).

Characteristics of the patients are summarized in Table 1. All patients experienced other symptoms of BD including oral ulcers (all patients), genital ulcers (5 subjects) and skin lesions (3 subjects) and arthritis (3 subjects). All patients experienced recurrent attacks of anterior uveitis with remissions and exacerbations and all had a history of posterior uveitis. There is no preoperative cataract in all eyes but cases no 4,5 and 7 developed cataract after vitrectomy. There was no history of trauma or macular hole in all 7 eyes.

**Table 1:** Clinical characters of the eyes with rhegamtougenous retinal detachment associated with Behçet’s disease

**Case no.**	**Age**	**Sex**	**Eye**	**Duration of Behçet’s disease (years)**	**RD topography**	**Duration of macular detachment**	**Associated signs**	**Previous history of IVTA**	**Visual acuity**				**Surgical technique**	**Postoperative follow-up (months)**
									**Pre operative**		**Post operative**			
									**Snellen**	**Logmar**	**Snellen**	**Logmar**		
1	32	Male	Right	5	Total RD	3 days	Vitritis	˗	FC	+2.00	6/18	0.5	Segmental SB	30
2	42	Male	Right	4	Temporal RD with Macula-off	10 days	Vitritis	+	HM	+3.00	3/60	1.3	Segmental SB	27
3	39	Male	Left	7	Lower RD with macula-off	7 days	BRVO	+	HM	+3.00	3/60	1.3	Encircling SB	16
4	30	Male	Left	3	Total RD	5 days	Panuveitis, PVR Grade C	˗	FC	+2.00	6/36	0.8	PPV	18
5	28	Female	Left	5	Total RD	5 days	PVR Grade B	˗	HM	+3.00	6/60	1.0	Initial treatment by segmental SB and PPV for recurrence	13
6	34	Male	Left	7	Total RD	8 days	BRVO	˗	FC	+2.00	6/60	1.0	Segmental SB	29
7	35	Male	Left	7	Combined TRD - RRD	14 days	BRVO, NV, VH	+	FC	+2.00	FC	2.0	PPV	21

RD = retinal detachment, VA = visual acuity, SB = scleral buckling, PPV = pars plana vitrectomy, PVR = proliferative vitreoretinopathy, IVTA = intravitreal triamcinolone acetonide, RD = retinal detachment, RRD = rhegamtougenous retinal detachment; TRD = tractional retinal detachment; FC = fingers counting; HM = hand motion; BRVO = branch retinal vein occlusion; NV = neovascularization, VH= vitreous hemorrhage.

In 3 patients there was a history of intravitreal injection of triamcinolone acetonide to treat attacks of panuveitis (2 eyes) or resistant cystoid macular edema (1 eye). All cases were injected in other clinics. In all 3 eyes, the detachment occurred within 8 weeks from the injection time. In one patient (case No. 4), the primary tear was masked by the concomitant posterior uveitis. In one patient (case No.7) there was combined tractional and rhegmatogenous RD secondary to old branch retinal vein occlusion (BRVO) with posterior small round tear just inside the upper arcade. In patients (1,2,4,5 and 6) single horseshoe tear was detected at or anterior to equator. , while in case No.3 multiple pre-equatorial retinal holes were found in two quadrants.

Five eyes were treated by conventional scleral buckling and 2 eyes were treated by PPV. Initial surgical reattachment was achieved in all seven eyes. In one eye (case No.5), the detachment recurred after SB due to occurrence of PVR. This patient was re-operated by PPV with successful reattachment.

The mean duration of follow up was 22.0 ± 6.7 months. In patients 4, 5 and 7, the oil was removed 6 months after vitrectomy. Cataract extraction via phacoemulsification with intraocular lens implantation was performed at the same time of oil removal, the retina remained attached in all eyes after silicone oil removal.

All patients received systemic and topical steroids prior to surgery. Mild to moderate anterior uveitis (+2 cells) and vitritis developed in cases no 2 and 6. Cystoid macular edema recurred in cases no 2 and 3 with the need to repeated triamcinolone injection 7 and 8 weeks postoperative respectively.

All patients had preoperative best corrected visual acuity of hand motion (HM) or finger counting (FC) and at the last follow up visual acuity improved in 6 (85.7%) eyes and did not change in one eye. Macular ischemia was observed in eyes with poor postoperative VA (case No.7).

## Discussion

Posterior segment involvement in BD often leads to irreversible and serious vision loss. Although most retinal manifestations of the disease have been reported in the literature, rhegmatogenous RD is rarely discussed in details. The current study presents a case series of patients with rhegmatogenous RD associated with BD with focus on the clinical course and visual outcome with surgical intervention.

The study describe herein showed that 85.7% of the patients were males with 85.7% of them less than 40 years of age. These findings are concordant with previous studies that have shown a male preponderance and it is generally associated with a more severe form of the disease in men, and a younger age of onset [1,3,4,6].

Previously, Akova et al. [14] reported the occurrence of rhegmatogenous RD in 3 eyes during the active stage of uveitis. Similarly in our cohort, one patient developed the detachment during the active stage of posterior uveitis and we confirm this possibility that uveitis may lead to retinal tear formation. In the current study, three patients (42.9%) had a history of IVTA within 8 weeks prior to the occurrence or rhegmatogenous RD. Therefore, we speculate IVTA may be a possible risk factor for retinal detachment. One possibility is that IVTA may induce posterior vitreous detachment with a risk to develop retinal tear at areas of abnormal vitreoretinal attachments. This agrees with Geck et al. [16] who reported the occurrence of PVD in 15 out of 61 eyes after intravitreal drug injection which may influence the outcome of underlying disease. Alternatively, the posterior uveitis itself may lead to vitreous gel shrinkage and development of tractional forces especially at areas with poor perfusion and the occurrence of rhegmatogenous RD. Therefore, patients who receive IVTA should be followed up cautiously after the injection for the risk of rhegmatogenous RD.

Primary scleral buckling was successful in 4 out of 5 patients (80%) during the follow up period. Only in one patient (case No. 5) scleral buckle failed due to PVR and retina was successfully reattached by PPV and silicone oil injection. In 2 patients (case No. 4 and No. 7) primary pars plana vitrectomy was done due to presence of PVR grade C (case No. 4) and combined tractional and rhegmatogenous RD (case No.7) with successful reattachment of the retina in both eyes. Therefore, Scleral buckling yielded good anatomical results in cases with simple retinal detachment especially in absence of PVR. Although some retina specialties may argue the value of buckling in these cases, it is still one successful option.

All patients received topical and systemic prednisolone in the perioperative period and tapered over a long period according to the clinical course, which we believe that it is an essential part of the therapy to avoid any exacerbations following surgery. The mean duration of postoperative follow-up in this study was considerable (22.0 ± 6.7 months) to address the surgical outcome, however larger studies are needed to address the long term outcome especially in BD where the recurrent exacerbations are expected to induce further complications.

The improvement in the VA was obtained in 6 out of 7 patients (85.7%), however the final visual outcome was limited. The reasons for that are the poor visual acuity at initial presentation, macular involvement in all cases and noticeable macular ischemia in eyes with poor visual outcome.

The current study had some limitations. First, the number of eyes with rhegmatogenous RD was relatively small. Second, there is limited information on the natural course of rhegmatogenous RD in such cases. Third, although some factors are thought to predispose to rhegmatogenous detachment, possibly panuveitis and IVTA, other possible precipitating factors need to be identified.

## Conclusion

In conclusion, even though rhegmatogenous RD is a rare posterior segment complication of BD but it should be kept in mind especially if the patient presented with marked diminution of vision on top of the chronic course of the disease and careful examination should be considered especially in cases with media opacity. Segmental scleral buckle or Pars Plana Vitrectomy are effective and save in management of this complication in BD patient with no unusual complications. Intravitreal injection of TA may be a precipitating factor for the development of rhegmatogenous RD so close observation of such patients after injection is crucial. Larger case series over longer follow up periods are needed to understand the natural course of the disease and accumulate more evidence on the surgical outcome.

## Competing interests

The authors declare that they have no competing interests.

## Authors’ contributions

SAD planned the study, conducted the data analysis and wrote the paper. MAG contributed to the planning of the study and revising of the paper. All surgical interventions were done by SAD. Both authors read and approved the final manuscript.

## Authors’ information

Manar A Ghali is co-author to this work.

## Pre-publication history

The pre-publication history for this paper can be accessed here:

http://www.biomedcentral.com/1471-2415/14/61/prepub
